# Caught in the Act: A Recurrent Tamponade After Coronary Artery Bypass Grafting With Culprit Lesion Identified on Computed Tomography Angiogram

**DOI:** 10.7759/cureus.49278

**Published:** 2023-11-23

**Authors:** Andrew J Gorton, Suresh Keshavamurthy, Conor Lowry, Michael E Sekela

**Affiliations:** 1 Department of Surgery, Division of Cardiothoracic Surgery, University of Kentucky, Lexington, USA; 2 Department of Radiology, University of Kentucky, Lexington, USA

**Keywords:** coronary artery bypass grafting (cabg), cardiac critical care, adult cardiac surgery, delayed postoperative cardiac tamponade, computed tomography angiography (cta)

## Abstract

Delayed cardiac tamponade after cardiac surgery is a rare complication with significant diagnostic challenges. The recurrence of cardiac tamponade physiology after initial intervention creates another degree of difficulty in the management of already medically complex patients. We present the case of a 65-year-old male who underwent four-vessel coronary artery bypass grafting that was complicated by the delayed presentation of cardiac tamponade requiring mediastinal exploration. Following this he developed a recurrence of cardiac tamponade with bleeding from a vein graft identified on multiphase spiral computed tomography angiography.

## Introduction

Cardiac tamponade is the accumulation of fluid around the heart with life-threatening hemodynamic changes [1]. If presenting early in the postoperative period, or less than 48 hours after surgery, it is considered acute. While delayed presentation or greater than 48 hours after cardiac surgery, it is characterized as subacute [2]. Delayed tamponade is a feared complication of cardiac surgery due to the difficulty in diagnosis and severe outcomes that warrant prompt treatment. The incidence of postoperative cardiac tamponade requiring reintervention has been reported as 0.1-6% in the case series [3]. Late tamponade (typically defined as >48h postoperatively) can be challenging to diagnose due to its more insidious onset [4]. Rapid diagnosis of this complication is essential and can involve imaging in addition to clinical findings. Surgical reintervention is often required for treatment [5].

## Case presentation

We present the case of a 65-year-old male with a past medical history of hypertension, hyperlipidemia, gastroesophageal reflux disease, and chronic obstructive pulmonary disease who was transferred to our institution for consideration for surgical revascularization. He presented to an outside institution with complaints of progressive dyspnea on exertion and was found to have multivessel coronary artery disease (CAD) on coronary angiography. At the time of transfer, he was also having significant diarrhea and was diagnosed with clostridium difficil colitis. Prior to surgical intervention, he was treated with a 10-day course of oral vancomycin with appropriate response. He then underwent four-vessel coronary artery bypass grafting (CABG) with left internal mammary artery (LIMA) to left anterior descending artery (LAD), reverse saphenous vein graft (SVG) to two obtuse marginal (OM) branch arteries of the left circumflex (LCx) in sequence, and SVG to the posterolateral branch of the right coronary artery (RCA).

**Figure 1:**
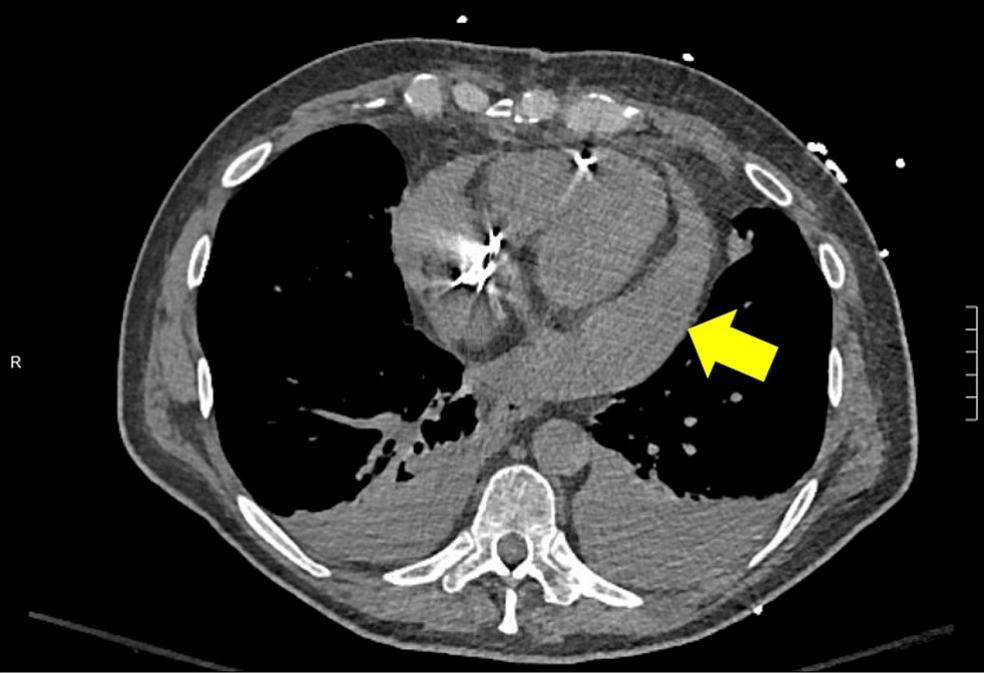
CT scan of the chest without contrast demonstrates dense moderate pericardial effusion (yellow arrow) with Hounsfield unit value consistent with acute hemopericardium.

His initial postoperative course was uncomplicated, and he progressed as expected. However, on postoperative day (POD) 4, he developed hypotension requiring fluid resuscitation and inotropic support. Computed tomography (CT) of the chest was performed demonstrating a moderate pericardial effusion with rightward bowing of the interventricular septum concerning cardiac tamponade (Figure 1).

He was taken to the operating room (OR) for mediastinal exploration and was found to have 300mL of serosanguinous fluid in the posterior pericardial space. There were no areas of active bleeding appreciated and he was returned to the intensive care unit (ICU) with stable hemodynamic status.

**Figure 2:**
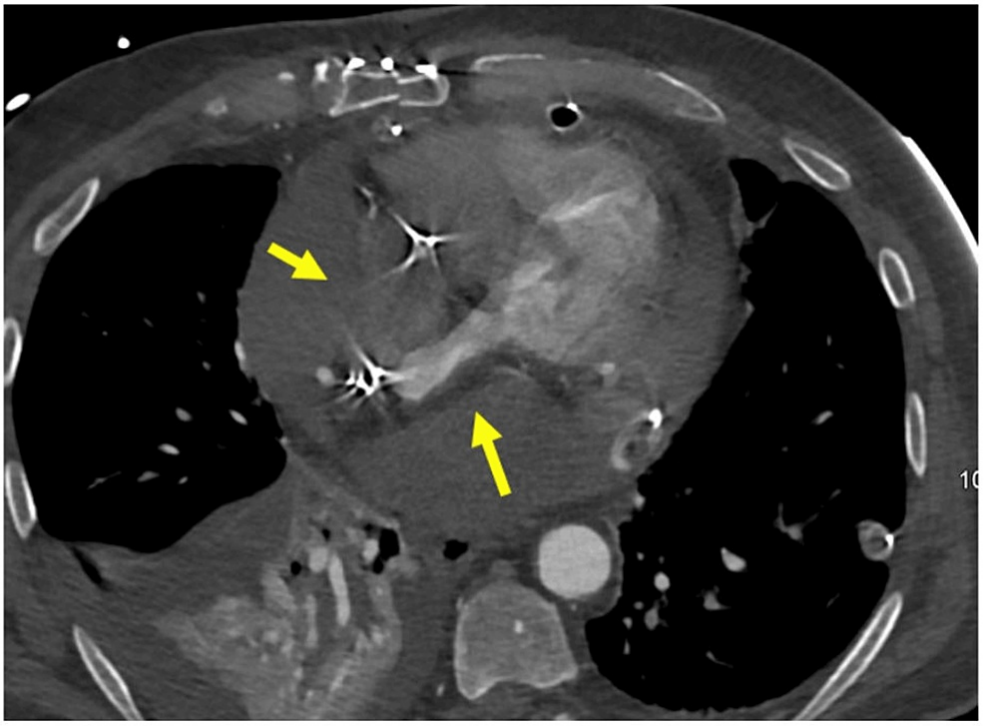
Arterial phase spiral CT angiogram showing acute tamponade with compression on the atria (yellow arrows).

**Figure 3:**
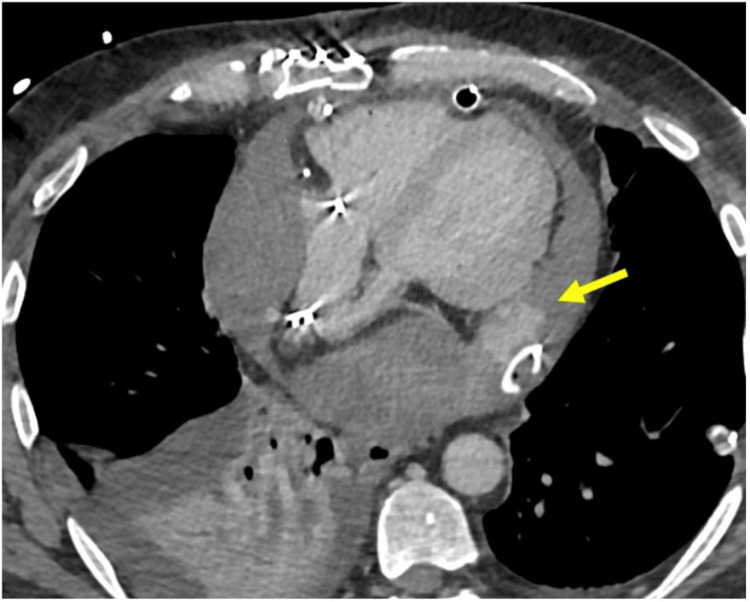
Delayed venous phase spiral CT angiogram showing active extravasation of contrast (yellow arrow) from a vessel on the posterior left ventricle, adjacent to the pericardial drain. This is more apparent compared to the arterial phase image at the corresponding axial slice.

He once again progressed well following surgery until POD 9 from his CABG and POD 5 from his re-exploration operation when he developed dyspnea, hypotension, and tachycardia. A pericardial drain had been left in place during his re-exploration with decreasing daily output. Due to the timing and clinical characteristics, the patient was sent for CT angiography (CTA) to assess for pulmonary embolism (PE) as well as other sources of clinical change. This imaging demonstrated acute tamponade due to compression of the atria in the arterial phase (Figure 2) and active extravasation of contrast from a vessel on the posterior left ventricle in the delayed venous phase (Figure 3).

He was returned to the OR for mediastinal re-exploration where blood clots were evacuated from the mediastinum and bleeding was noted from the SVG to OM. This was controlled with a single horizontal mattress suture and all grafts remained patent on examination.

After this point, he progressed with standard postoperative care and in-hospital rehabilitation services. He was discharged to home 21 days after his initial CABG.

## Discussion

This case demonstrates two important considerations in the post-cardiotomy patient; first that cardiac tamponade may have a delayed and insidious onset and second that CTA may be used as a modality for assessment of coronary bypass grafts.

Delayed cardiac tamponade following cardiac surgery was described in two case series from 1968 [6] and 1972 [7]. Interestingly, all of the first seven cases were in pediatric patients having undergone the Waterston operation for cyanotic heart disease (anastomosis of the aorta and right pulmonary artery). The later case series identified the complication in other operations, but again in pediatric patients. In these series, the range of presentation was from two weeks postoperatively to a single case presenting five weeks after surgery. At this point in the history of cardiac surgery, this was presented as evidence that simply keeping patients in the hospital longer following surgery would not prevent such complications. These series in pediatric patients did not identify a single cause for the presentation and surgical technique was ruled out in most patients.

Compounding the difficulty in diagnosing delayed cardiac tamponade after cardiac surgery is the limited utility of transthoracic echocardiography (TTE), which is considered a first-line diagnostic tool. TTE was highly recommended for evaluation of pericardial effusion by the American College of Cardiology (ACC), American Heart Association (AHA), and American Society of Echocardiography (ASE) in 2003 guidelines [8]. The European Society of Cardiology (ESC) 2015 guidelines recommend TTE as the initial imaging modality for the assessment of pericardial effusion and hemodynamic impact [9]. Unfortunately, in post-cardiotomy patients TTE can be quite challenging, and pericardial effusion may not be visualized. One study evaluated echocardiography and CT imaging of the thorax in post-cardiotomy patients with suspected cardiac tamponade [10]. In this 25-patient study, TTE was found to have sensitivity, specificity, positive predictive value, and negative predictive value of 75%, 64%, 75%, and 64% respectively for pericardial effusion using CT imaging as the reference standard. They concluded that with the limited diagnostic accuracy of TTE consideration should be given to CT imaging in suspected cases of delayed cardiac tamponade.

Multiple studies have demonstrated that cardiac CTA performed after cardiac surgery can be utilized for the assessment of coronary artery graft patency and that incidental findings with potential clinical significance are common. One such study examined 259 patients undergoing cardiac CTA following CABG at a mean of 5.2 days after surgery [11]. Within this study, 19.7% were found to have incidental findings with potentially significant effects. These included noncardiac findings including lung cancer and PE, cardiac findings of perfusion defects and intracardiac thrombi, and surgical complications including bypass graft occlusion in 6.6% of patients. A separate study utilized CTA to evaluate graft patency at one year following CABG [12]. The overall rate of graft failure was 7.2% in this 134-patient study with 10.8% of venous grafts and 0.7% of arterial grafts exhibiting new failure. Those patients with graft failure had higher rates of recurrent angina and need for revascularization. Whether routine CTA should be performed following CABG was addressed in a study of 305 patients following CABG [13]. They identified graft issues in 15% of patients and additional incidental findings in 44% of patients. These findings were most commonly pleural effusion and pneumothorax with smaller incidence of pericardial effusion, cardiac thrombus, and sternal dehiscence. Many of these incidental findings affected further postoperative management. While limited by the retrospective nature and small population size of the studies it lends support to consideration of routine postoperative CTA in CABG patients.

## Conclusions

Delayed cardiac tamponade following cardiac surgery is a rare and potentially devastating complication. A high level of clinical suspicion, expedited workup, and aggressive management should be employed to optimize patient outcomes. This case and previous studies have demonstrated the utility of CTA in evaluating pericardial effusion and coronary bypass graft patency in patients following CABG.
